# EOR-1 mediates non-cell autonomous regulation of abts-1 gene expression in HSNs​

**DOI:** 10.17912/pm1b-9z95

**Published:** 2018-07-12

**Authors:** Yoichi Shinkai, Motomichi Doi

**Affiliations:** 1 Molecular Neurobiology Research Group and DAILAB, Biomedical Research Institute, National Institute of Advanced Industrial Science and Technology (AIST), Tsukuba Central 6, 1-1-1, Higashi, Tsukuba, Ibaraki, 305-8566, Japan

**Figure 1. Non-cell autonomous regulation of abts-1b expression in HSNs.**
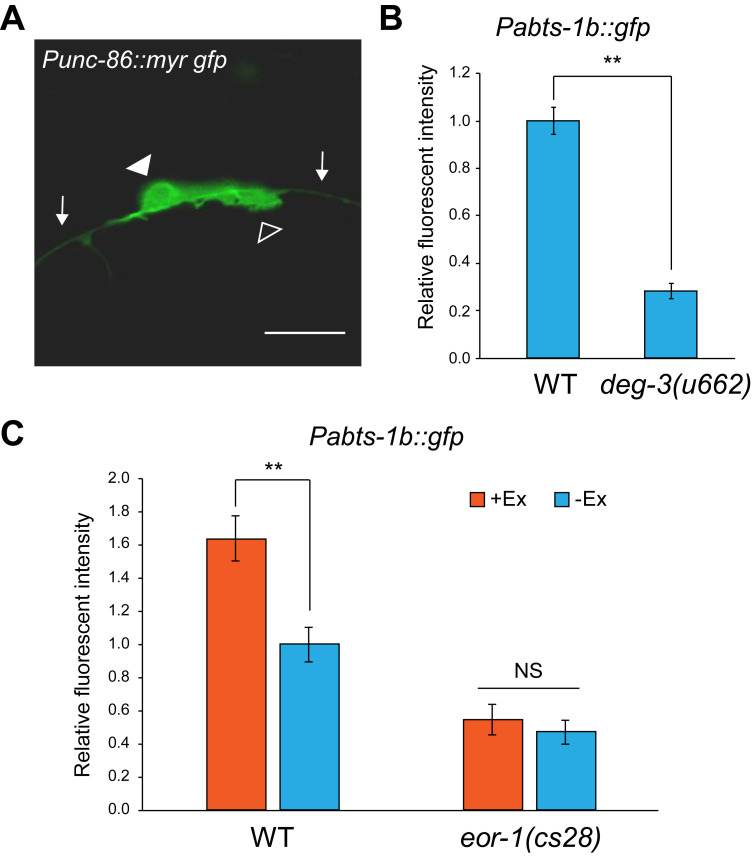
(A) A representative image of an HSN at L3 larval stage, obtained from strain CX5974. The closed arrowhead indicates the cell body of an HSN and the open arrowhead indicates an elongating neurite from the cell body. Arrows indicate the axon of PLM neuron. White scale bar indicates 10 mm. (B) Comparison of *Pabts-1b::gfp* expression in HSNs between wild-type and *deg-3(u662)* mutant worms at the adult stage. Error bars indicate SEM (n ≥ 49, **p < 0.01, Mann-Whitney U test). (C) *Pabts-1b::gfp* expression in HSNs was analyzed in worms expressing PKC-1(gf) under the control of the *mec-7* promoter. Data from wild-type or *eor-1(cs28)* mutant worms are shown. Worms carrying the transgene (+Ex) and worms not carrying the transgene (−Ex) were compared. Error bars indicate SEM (n ≥ 22, **p < 0.01, Mann-Whitney U test)

## Description

As neurons mature, responses to γ-aminobutyric acid (GABA) are switched from excitation to inhibition. This excitatory-to-inhibitory GABA functional switch is triggered by GABA-induced depolarization of neurons (Ganguly et al. 2001). Such GABA-induced neuronal depolarization upregulates the expression of chloride exporters, which leads to a decrease in intracellular chloride concentration, but the detailed mechanisms still remain unknown.

In *Caenorhabditis elegans* (*C. elegans*), several chloride exporters have been described (Tanis et al. 2009; Bellemer et al. 2011). Among them, *abts-1* encodes a sodium-driven chloride-bicarbonate transporter and is expressed in many neurons, including hermaphrodite-specific neurons (HSNs) that regulate egg-laying behavior in mature hermaphrodites. Thus, *abts-1* mutants show a resistant phenotype in GABA receptor (GABAR) agonist-induced inhibition of egg laying because of a defect in the GABA functional switch (Bellemer et al. 2011).

In the present study, we investigated whether *abts-1* expression in HSNs is dependent on neuronal activities through synaptic transmission. *abts-1* is expressed in HSNs from the L4 larval stage onward (Shinkai et al. 2018). To measure *abts-1* expression levels in HSNs, we used transgenic worms carrying *vsIs138 [Pabts-1b::gfp, lin15(+)]*. Quantitative fluorescent measurements were performed as previously described (Shinkai et al. 2018). Since presynaptic inputs are important for the GABA functional switch, we investigated the effect of PLM neurons, which innervate to HSNs (White et al. 1986). Importantly, PLM touch receptor neurons show contact with HSNs during HSN development (Figure 1A), suggesting that they may have a possible role in neuronal maturation of HSNs. Therefore, we measured the expression level of *Pabts-1b::gfp* in HSNs of *deg-3(u662)* mutants, which lose touch sensory neurons including PLM neurons at the L2-L3 larval stage because of abnormal cell death (Treinin and Chalfie 1995). *Pabts-1b::gfp* expression was significantly reduced in HSNs of *deg-3(u662)* mutants (Figure 1B), suggesting that presynaptic inputs from PLM neurons are required for the expression of *Pabts-1b::gfp*. Next, we investigated the effect of increased presynaptic input from PLM neurons. The gain-of-function mutation of *pkc-1* has been previously used to enhance presynaptic activities through an increase of both synaptic and dense-core vesicle release from neurons of interest (Sieburth et al. 2005; Sieburth et al. 2007; Shinkai et al. 2011; Inoue et al. 2013). Compared to wild-type worms, the transgenic worms expressing *pkc-1(gf)* in PLM neurons showed increased expression of *Pabts-1b::gfp* in HSNs (Figure 1C). These results suggest that synaptic inputs from presynaptic neurons including PLM modulate the expression of *Pabts-1b::gfp* in HSNs in a non-cell autonomous manner. We recently reported that EOR-1 transcription factor is involved in the chromatin-state regulation of *abts-1b* promoter region at an early developmental stage, thus preparing for subsequent gene expression for the maturation of HSNs (Shinkai et al. 2018). To uncover the relationship between chromatin alteration by EOR-1 and presynaptic input-dependent regulation of *Pabts-1b::gfp* expression during HSN neuronal maturation, we analyzed whether the effect of *Pmec-7::pkc-1(gf)* on *Pabts-1b::gfp* expression is altered in *eor-1* mutants. *eor-1* mutants did not show enhancement of *Pabts-1b::gfp* expression by *pkc-1(gf)* (Figure 1C), suggesting that EOR-1 is required for non-cell autonomous regulation of the *Pabts-1b::gfp* expression in HSNs.

In summary, we provided evidence for activity-dependent regulation of *abts-1* gene expression during HSN neuronal maturation and propose that EOR-1-mediated chromatin alterations in the *abts-1* promoter may be prerequisites for non-cell autonomous regulation of *abts-1* gene expression in HSNs.

## Reagents

The strains used in this study were as follows: Wild-type strain N2, *eor-1(cs28)*, *deg-3(u662)*, LX1514 (*vsIs138 [Pabts-1b::*gfp*, lin-15(+)]*), and CX5974 (*kyIs262 [unc-86::*myr gfp *+ Podr-1::*rfp*]*). All strains were cultured on NGM plates with *E. coli* strain OP50 as a food source under standard conditions.

The transgenes used in this study were as follows:*vsIs138 [Pabts-1b::*gfp*, lin-15(+)]*, *Ex[Pmec-7::pkc-1(gf)* SL2 mCherry*, Pmyo-2::*gfp*]*, and *kyIs262 [unc-86::*myr gfp *+ odr-1::*rfp*].*

## References

[R1] Bellemer A, Hirata T, Romero MF, Koelle MR (2011). Two types of chloride transporters are required for GABA(A) receptor-mediated inhibition in C. elegans.. EMBO J.

[R2] Ganguly K, Schinder AF, Wong ST, Poo M (2001). GABA itself promotes the developmental switch of neuronal GABAergic responses from excitation to inhibition.. Cell.

[R3] Inoue A, Sawatari E, Hisamoto N, Kitazono T, Teramoto T, Fujiwara M, Matsumoto K, Ishihara T (2013). Forgetting in C. elegans is accelerated by neuronal communication via the TIR-1/JNK-1 pathway.. Cell Rep.

[R4] Shinkai Y, Kuramochi M, Doi M (2018). Regulation of chromatin states and gene expression during HSN neuronal maturation is mediated by EOR-1/PLZF, MAU-2/cohesin loader, and SWI/SNF complex.. Sci Rep.

[R5] Shinkai Y, Yamamoto Y, Fujiwara M, Tabata T, Murayama T, Hirotsu T, Ikeda DD, Tsunozaki M, Iino Y, Bargmann CI, Katsura I, Ishihara T (2011). Behavioral choice between conflicting alternatives is regulated by a receptor guanylyl cyclase, GCY-28, and a receptor tyrosine kinase, SCD-2, in AIA interneurons of Caenorhabditis elegans.. J Neurosci.

[R6] Sieburth D, Ch'ng Q, Dybbs M, Tavazoie M, Kennedy S, Wang D, Dupuy D, Rual JF, Hill DE, Vidal M, Ruvkun G, Kaplan JM (2005). Systematic analysis of genes required for synapse structure and function.. Nature.

[R7] Sieburth D, Madison JM, Kaplan JM (2006). PKC-1 regulates secretion of neuropeptides.. Nat Neurosci.

[R8] Tanis JE, Bellemer A, Moresco JJ, Forbush B, Koelle MR (2009). The potassium chloride cotransporter KCC-2 coordinates development of inhibitory neurotransmission and synapse structure in Caenorhabditis elegans.. J Neurosci.

[R9] Treinin M, Chalfie M (1995). A mutated acetylcholine receptor subunit causes neuronal degeneration in C. elegans.. Neuron.

[R10] White JG, Southgate E, Thomson JN, Brenner S (1986). The structure of the nervous system of the nematode Caenorhabditis elegans.. Philos Trans R Soc Lond B Biol Sci.

